# SOCS2 Inhibits Mitochondrial Fatty Acid Oxidation via Suppressing LepR/JAK2/AMPK Signaling Pathway in Mouse Adipocytes

**DOI:** 10.1155/2020/3742542

**Published:** 2020-07-13

**Authors:** Tiantian Zhang, Yizhe Chen, Jiarui Cai, Miao Pan, Qian Sun, Jing Zhang, Chao Sun

**Affiliations:** Key Laboratory of Animal Genetics, Breeding and Reproduction of Shaanxi Province, College of Animal Science and Technology, Northwest A&F University, Yangling, Shaanxi 712100, China

## Abstract

Suppressor of cytokine signaling 2 (SOCS2) plays an important role in fat deposition, skeletal muscle, central nervous system development, and mitochondria biogenesis. Nevertheless, the regulatory mechanisms of SOCS2 on mitochondrial fatty acid oxidation (FAO) remain unclear. Leptin could inhibit food intake and increase thermogenesis through leptin receptor (LepR), which was present in the hypothalamus and certain peripheral organs, including adipose tissue. With strong interest, we focused on the connection between leptin and SOCS2 and their effect on FAO in adipocytes. In our study, we found that the mRNA level of SOCS2 and the protein levels of PGC-1*α*, CPT-1b, FAT, and p-ACC were elevated by leptin in the inguinal adipose tissue of mice. On the contrary, the protein levels of FABP4, FATP1, and FAS were declined. The genes related to fatty acid oxidation such as PGC-1*α*, NRF-1, TFAM, CPT-1b, AOX1, COX2, and UCP2 were attenuated by SOCS2, but elevated by leptin. Moreover, fatty acid oxidation enzyme MCAD, LCAD, and Cyt C levels were reduced in response to SOCS2. These reductions correspond well with the reduced release of free fatty acid and the reduction of mitochondrial complexes I and III by SOCS2. Furthermore, JAK2/AMPK pathway-specific inhibitors could block the mitochondrial FAO; hence, this pathway was implied to have a potential impact on FAO. Together, these studies suggested that SOCS2 had a negative effect on mitochondrial fatty acid oxidation, and the LepR/JAK2/AMPK pathway played a crucial role in this process.

## 1. Introduction

The suppressor of cytokine signaling (SOCS), as an inhibitor of cytokine signaling pathways, downregulates the signal pathway Janus kinase/signal transducers and activators of transcription (JAK/STAT) in several interconnected mechanisms [1]. Our previous research showed SOCS3 inhibited the JAK2/STAT3 signaling pathway and promoted inflammation and apoptosis in the adipose tissue and liver [2, 3]. SOCS2, another member of the SOCS family, is widely expressed in the muscle, nerve, pancreas, and adipose tissues [4–6]. Several studies also indicated that SOCS2 has important actions in numerous physiological processes, such as fat deposition, skeletal muscle development, central nervous system development, metabolism, immune response, mammary gland development, cancer, and other cytokine-dependent processes [7–11]. Our previous research showed that SOCS2 was an important negative regulator of GH signaling in porcine adipocytes (promotion of adipogenesis and inhibition of lipolysis) and inhibited mitochondria biogenesis in C2C12 cells [2, 6]. Hence, we are interested to know if SOCS2 could regulate fatty acid oxidation in mitochondria.

Leptin, an adipocyte-derived hormone, controls energy balance and body weight via binding to leptin receptor (LepR) [12]. Leptin receptor, a member of cytokine receptor family, can activate the JAK tyrosine kinases (JAK1, JAK2, JAK3, and Tyk2) phosphorylation and strengthen the STAT transcription factors [13, 14]. And it can also be phosphorylated by JAK2 in turn [12]. SOCS3 is demonstrated as a major negative regulator of both leptin and insulin signaling, and SOCS3 overexpression in muscle also suppresses leptin-regulated genes involved in fatty acid oxidation and mitochondrial functions [15, 16]. Steinberg GR et al. [17, 18] reported that SOCS3 could mediate skeletal muscle leptin resistance which may contribute to the aberrant regulation of fatty acid metabolism observed in obesity. SOCS2 was also reported to interact with LepR [19]. However, the function of this interaction in different tissues needs to be further elucidated.

AMP-activated protein kinase (AMPK) is a key molecule in energy homeostasis and acts as an important downstream effector of leptin signaling. Chronic administration of leptin increases the expression of AMPK in the skeletal muscle [18]. Leptin- or LepR-deficient rodents show a decreased AMPK activity in the liver [20]. Leptin activates the AMPK pathway and stimulates fatty acid oxidation by blocking the effect of acetyl-CoA carboxylase (ACC) in the skeletal muscle [21].

Mitochondrial fatty acid oxidation (FAO) is the major pathway for the degradation of fatty acids and plays a pivotal role in generating ATP in various tissues [22]. FAO is related with a series of enzymes, transporters, and other facilitating proteins, such as fatty acid transport protein 1 (FATP1), carnitine palmitoyl transferase I-b (CPT1b), medium-chain acyl-CoA dehydrogenase (MCAD), and long-chain acyl-CoA dehydrogenase (LCAD), which carry out the FA cellular uptake, mitochondria shuttle, and the *β*-oxidation steps [23–26].

In our study, we contrasted the different roles of SOCS2 and leptin in fatty acid oxidation and further investigated the mechanism of SOCS2 on mitochondrial fatty acid oxidation in mouse adipocytes. These data were devoted to contributing to fundamental research of energy homeostasis and the prevention and treatment of related metabolic diseases.

## 2. Materials and Methods

### 2.1. Animal Experiment

Based on the guidelines and regulations, all animal experimental procedures were approved by the Animal Ethics Committee of Northwest A&F University (Yangling, Shaanxi). Three-week-old C57BL/6J male mice were purchased from the Laboratory Animal Center of the Fourth Military Medical University (Xi'an, China). All animals were bred on-site at the College of Animal Science and Technology, Northwest A&F University. They were allowed *ad libitum* access to water and standard laboratory mice chow. An animal room was maintained under controlled conditions of temperature at 25°C ± 1°C, humidity at 55 ± 5%, and a 12 h-light/12 h-dark cycle.

For the leptin administration study, mice (all mice are male) from four-week age were individually caged. After daily body weight and temperature and food intake measurement, murine leptin (PeproTech) (1 mg/kg body weight) or saline was injected intraperitoneally from six-week to eight-week age, which were carried out at around 9:00 am every day. The *in vivo* experiments were performed after 7 days of leptin infection. The recombinant adenovirus overexpression vector of SOCS2 (Ad-SOCS2) and recombinant adenovirus interference vector of SOCS2 (sh-SOCS2) had been constructed in our lab before [27]. Vehicle or Ad-SOCS2 and sh-SOCS2 were administered as a daily intraperitoneal injection for 7 days. The purified adenovirus titer is 10^10^ PFU, and the injection volume of each mouse is 30 *μ*L/d. After two hours of the last injection, mice in various groups were sacrificed using overdosed ethyl ether. Immediately, the inguinal white adipose tissue (iWAT) was dissected and kept for the studies as follows.

### 2.2. Primary Adipocyte Culture

The preadipocyte culture was carried out according to our previous publication [28]. In short, iWAT were dissected from 6-week-old male C57BL/6J mice, and visible connective fibers and blood vessels were removed and washed three times with PBS containing 200 U/mL penicillin (Sigma, St. Louis, USA) and 200 U/mL streptomycin (Sigma, St. Louis, USA). Then, the iWAT was finely minced (1 mm^3^) with scissors and incubated in 10 mL digestion buffer containing Dulbecco's modified Eagle medium (DMEM)/F12 (Gibco, USA), 100 mM HEPES (Sigma, St. Louis, USA), 15% bovine serum albumin (Sigma, St. Louis, USA), and 2 mg/mL Type I collagenase (Sigma, St. Louis, USA) in a shaking water bath at 37°C for 50 min. The digested tissue was filtered through nylon screens with 250 *μ*m and 70 *μ*m mesh openings to remove undigested tissues and large cell aggregates. The cell pellets were centrifuged at 800× g for 7 min at room temperature to separate floating adipocytes from cell pellets. Then, suspend and wash the cell pellets twice with culture media (DMEM/F12 with 15% bovine serum albumin and 100 nM HEPES), and these stromal vascular cells were seeded into 35 mm culture dishes at a density of 8 × 10^4^ cells/dish and incubated at 37°C under a humidified atmosphere of 5% CO_2_ and 95% air until confluence. The medium was changed every other day. After reaching 95% confluence, cells were induced to differentiate using DEME/F12 with 10% FBS and 100 nM insulin for 5-6 days until exhibiting a massive accumulation of fat droplets [29].

### 2.3. Cell Viability Assay

Cell viability was determined by using Cell Counting Kit 8 (CCK-8, Vazyme, China) assay according to the instructions. The transfected cells were seeded in a 96-well plate at a density of 2 × 10^5^ and cultured for 12 h. 10 *μ*L CCK-8 solution was added into each well and incubated for 1 hour at 37°C. Absorbance was quantified at 450 nm by Vector 5 (Bio-Tech Instruments, USA).

### 2.4. Fatty Acid Oxidation Measurement

Palmitate oxidation to CO_2_ and the incorporation of palmitate into lipids were measured according to a previously published method [30, 31]. Adipocytes were washed in Krebs-Ringer bicarbonate HEPES buffer (KRBH buffer: 135 mM NaCl, 3.6 mM KCl, 0.5 mM NaH_2_PO_4_, 0.5 mM MgSO_4_, 1.5 mM CaCl_2_, 2 mM NaHCO_3_, and 10 mM HEPES, pH 7.4) that contained 0.1% BSA, preincubated at 37°C for 30 min in KRBH 1% BSA, and washed again in KRBH 0.1% BSA. Cells were then incubated for 3 h at 37°C with fresh KRBH containing 1 *μ*Ci/mL [1-^14^C] palmitate (Perkin Elmer, USA) bound to 1% BSA. Oxidation measurements were performed by trapping the radioactive CO_2_ in a parafilm-sealed system. The reaction was stopped by the addition of 40% perchloric acid through a syringe that pierced the parafilm. For an *in vivo* study of palmitate oxidation to CO_2_ and the incorporation of palmitate into different tissues, we used the modified method from Buettner et al. [32]. Plasma and cell triglycerides (TG) and free fatty acid (FFA) contents were determined by using commercial ELISA kits (Jiancheng, China).

### 2.5. Mitochondrial Respiratory Activity

Adipocyte mitochondria were isolated using the Cell Mitochondria Isolation kit (Beyotime, China). Cells were harvested and washed with cool-PBS twice and then suspended in the ice-cold isolation buffer for 15 min. After the cells were homogenized, the homogenate was centrifuged at 1,000× g for 10 min at 4°C. The supernatant was collected and centrifuged at 11,000× g for 10 min at 4°C. The mitochondria were collected in the sediments. The activities of the mitochondrial complexes were determined using the Mito Complex I and III Activity Assay kits (GenMed Scientifics Inc., China).

### 2.6. Mitochondrial Content and Mitochondrial Damage Assay

Fluorescent probe JC-1 (Beyotime, China) was used to estimate mitochondrial membrane potential. Briefly, cells were incubated with 5 *μ*g/mL JC-1 at 37°C for 10 min, then washed twice with PBS and placed in fresh medium without serum. Images of the cells were scanned by a Fluorescence Microscope (Nikon TE2000-U, Japan). At the same time, cells were gently harvested with trypsin and transferred on ice to the flow cytometer. JC-1 was excited at 488 nm, and the monomer signal (green) was recorded at 525 nm (JC-1 monomer) on a flow cytometer using a minimum of 10,000 cells per sample. Simultaneously, the aggregate signal (red) was recorded at 590 nm (JC-1 aggregates). The ratio of red/green fluorescent intensity was calculated.

Cyt C immunofluorescence analysis was performed 48 h after plasmids transfection as previously described [33]. Cells were washed three times with PBS and fixed with 10% neutral formalin for 30 min and washed with PBS, then incubated with the rabbit against rat Cyt C antibody (Boster Biological Technology Co., China) (diluted 1 : 100 in PBS) for 12 h at 4°C. After the incubation, cells were washed twice with PBS for 3 min and then incubated with fluorescein isothiocyanate-conjugated goat against rabbit IgG antibody (Boster, China) (diluted 1 : 100 in PBS) for 1 h at room temperature and washed again in PBS. Finally, the cells were illuminated with the appropriate laser line and photographed with a TE2000 Nikon fluorescence microscope (excitation filter BP 450-490, a beam splitter FT510 and an emission filter LP520, Tokyo, Japan).

Relative amounts of mtDNA copy number and nuclear DNA copy number were detected using a QPCR method. Pairs of primers for the COX2 mtDNA and nDNA 18S rRNA were from our laboratory. The QPCR system was performed according to the instructions (COX2 forward 5′⟶3′: GGGAAGCCTTCTCCAACC; COX2 reverse 5′⟶3′: GAACCCAGGTCCTCGCTT). After treatment with the indicated plasmids, ATP concentration was determined using the Luciferase-based ATP-assay kit (Roche, Switzerland).

### 2.7. Cell Lipid Measurement

The Bodipy 493/503 staining (Life Technology, CA) was used to visualize lipid droplet in the adipocytes transfected with indicated plasmids. In brief, cells were seeded in a 96-well plate at a density of 5 × 10^3^ and cultured for 12 h. Then, cells were washed in PBS buffer, fixed in 4% paraformaldehyde for 30 min. Bodipy dye was diluted in PBS buffer to the final concentration of 1 mg/mL and applied to cells for 15 min. Then DAPI (4′,6-diamidino-2-phenylindole) solution (10 *μ*g/mL) was added and incubated for 30 min. Digital images were obtained with a Nikon TE2000-U Fluorescence Microscope (Tokyo, Japan). The area of the stained cells with Bodipy and the droplet diameter frequency distributions was measured by Image-Pro Plus analyzer (Media Cybernetics, MD) in 10 random microscopic fields (×40).

### 2.8. Enzyme-Linked Immunosorbent (ELISA) Assay

The contents of fatty acid transporters and MCAD and LCAD levels were determined using commercial ELISA kits (R&D Systems, USA). Adipose tissues were collected as described previously. The cells were collected after Ad-SOCS2 and sh-SOCS2 transfection and then disrupted by ultrasonication (28 kHz, 30 min).

### 2.9. Real-Time PCR Analysis

Total RNA was extracted from cells by using RNAiso Plus Reagent (TaKaRa, Dalian, China) and used for synthesizing of the first-strand cDNA with PrimeScript® RT reagent Kit (TaKaRa, Dalian, China). Primers for *SOCS1*, *SOCS2*, *SCOS3*, *LepR*, *PGC-1α*, *NRF-1*, *TFAM*, *AOX1*, *COX2*, *UCP2*, and *GAPDH* were designed by Premier 5.0 software. *GAPDH* was used as the internal control. Real-time PCR was performed with an iQ5 system (Bio-Rad, USA) using a 20 *μ*L reaction mixture containing 12.5 *μ*L SYBR *Premix Ex Taq*™ II (TaKaRa, Japan), 1 *μ*L forward primer, 1 *μ*L reverse primer, 2 *μ*L template cDNA, and 8.5 *μ*L ddH_2_O. A 2^−*ΔΔ*Ct^ method was chosen to analysis the data (ΔCt = Ctwas for target gene and Ct for reference gene, andΔΔCt = ΔCtwas for the treatment group and –*ΔΔ*Ct for the control group).

### 2.10. Western Blotting Analysis

Western blotting analysis was performed as previously described [34]. Mouse adipocytes were solubilized in adipocyte lysing buffer and incubated for 40 min at 4°C, then the solution was centrifuged at 12,000× g for 15 min at 4°C, and the supernatants were used for determination of protein concentration. Protein samples (30 *μ*g) were separated by electrophoresis on 12% and 5% SDS-PAGE gels using slab gel apparatus and transferred to PVDF nitrocellulose membranes (Millipore, USA), blocked with 5% skim milk powder/Tween 20/TBST at room temperature for 2 h. The membranes were then incubated with primary antibodies against SOCS2, CPT-1b, p-ACC, ACC1, p-JAK2, JAK2, p-AMPK, AMPK, PGC-1*α*, and GAPDH (Bioworld, China) at 4°C overnight. Following this step, the appropriate HRP-conjugated secondary antibodies (Baoshen, China) were added and incubated for 2 h at room temperature. Proteins were visualized using chemiluminescent peroxidase substrate (Millipore, USA), and then, the blots were quantified using a ChemiDoc XRS system (Bio-Rad, USA).

### 2.11. Statistical Analysis

Statistical analyses were conducted using SAS v8.0 (SAS Institute, Cary, NC). Data were analyzed using either one-way ANOVA or two-way ANOVA depending on the number of variables. Comparisons among individual means were made by Fisher's least significant difference (LSD). Data were presented as the mean ± SD. Difference was considered statistically significant (^∗^*P* < 0.05, ^∗∗^*P* < 0.01).

## 3. Results

### 3.1. Leptin Promoted Fatty Acid Oxidation and SOCS2 mRNA Level in Mouse Adipose Tissue

**Figure 1:**
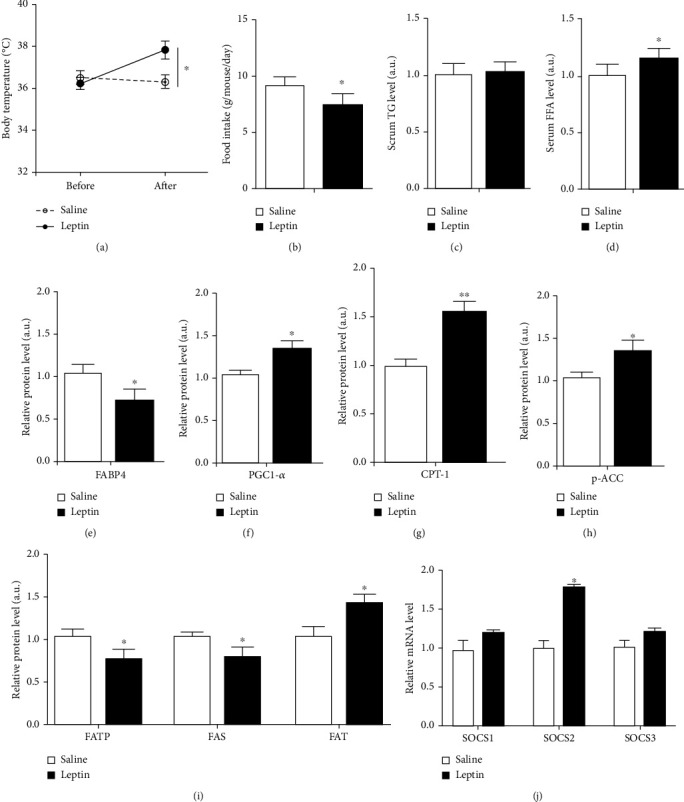
The specific increase of SOCS2 in adipose tissue after leptin administration. Mice were intraperitoneally injected with leptin (1 mg/kg body weight) or saline. (a) Rectal temperature of male mice was measured after leptin or saline treatment (*n* = 6). (b). Effect of leptin on food intake. Food intake was measured daily during leptin injection study (*n* = 6). (c and d) Serum TG and FFA levels of mice after leptin or saline injection (*n* = 6). Effects of leptin on FABP4 (e), PGC-1*α* (f), CPT-1b (g), p-ACC (h), and FATP1, FAS, and FAT (i). Protein expression levels of inguinal adipose tissue after leptin or saline injection (*n* = 4). (j) mRNA levels of SOCS1, SOCS2, and SOCS3 of inguinal adipose tissue after leptin or saline injection (*n* = 4). All the protein levels (e–i) were detected by the ELISA test. Values are the means ± SD. ^∗^*P* < 0.05, ^∗∗^*P* < 0.01.

Figures 1(a) and 1(b) indicate that leptin injection increased body temperature (*P* < 0.05) and reduced food intake (*P* < 0.05) in mice. Meanwhile, leptin elevated the serum FFA level (*P* < 0.05), but not triglycerides (TG) (*P* > 0.05) (Figures 1(c) and 1(d)). Fatty acid-binding protein 4 (FABP4) protein level was reduced after leptin treatment (*P* < 0.05, Figure 1(e)). Conversely, the protein levels of peroxisome proliferator-activated receptor gamma coactivator 1-alpha (PGC-1*α*) (*P* < 0.05), carnitine palmitoyl transferase I-b (CPT-1b) (*P* < 0.01), and p-ACC (*P* < 0.05) were increased as shown in Figures 1(f)–1(h). Fatty acid transport protein 1 (FATP1) and fatty acid synthase (FAS) protein levels were declining. Conversely, fatty acid translocase (FAT) level increased (*P* < 0.05, Figure 1(i)). Furthermore, we found leptin injection increased SOCS2 mRNA level (*P* < 0.05, Figure 1(j)).

### 3.2. SOCS2 Reversed the Promoting Function of Leptin on Fatty Acid Oxidation

**Figure 2:**
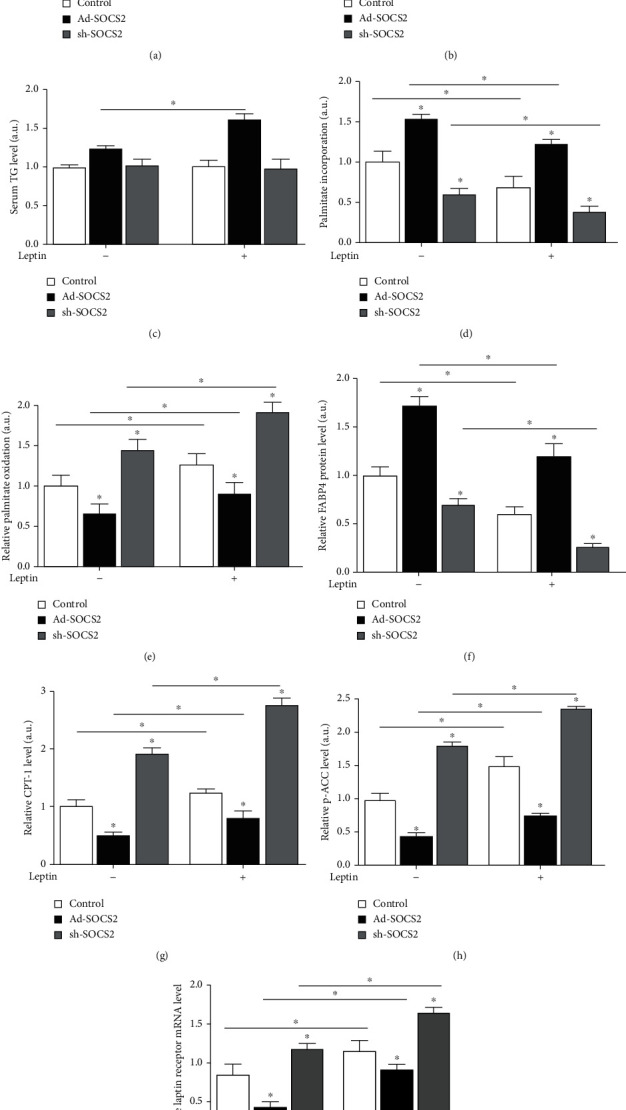
SOCS2 reversed the promoting function of leptin on fatty acid oxidation. Eight-week-old male mice were intraperitoneally injected with leptin (1 mg/kg body weight) or saline. (a) SOCS2 mRNA level in iWAT after Ad-SOCS2 or sh-SOCS2 injection and leptin injection into mice (*n* = 4). Serum FFA (b) and TG (c) levels of mice after Ad-SOCS2 or sh-SOCS2 injection and leptin injection (*n* = 4). (d) Palmitate incorporation into iWAT after Ad-SOCS2 or sh-SOCS2 injection and leptin injection (*n* = 4). (e) Relative palmitate oxidation to CO_2_ after Ad-SOCS2 or sh-SOCS2 injection and leptin injection in iWAT (*n* = 4). Protein expression levels of FABP4 (f), CPT-1b (g), and p-ACC (h) in iWAT after Ad-SOCS2 or sh-SOCS2 injection and leptin injection (*n* = 4). (i) Leptin receptor mRNA level in iWAT after Ad-SOCS2 or sh-SOCS2 injection and leptin injection (*n* = 4). All the protein levels (f–h) were detected by the ELISA test. Values are the means ± SD. ^∗^*P* < 0.05, ^∗∗^*P* < 0.01.

To clarify the function of SOCS2 on fatty acid oxidation, *in vivo* gain or loss of function of SOCS2 was performed by intraperitoneal injection of recombinant adenovirus vector Ad-SOCS2 or sh-SOCS2. The following data provide evidence that the effects of leptin and SOCS2 are preserved in adipocytes. Our results indicated Ad-SOCS2 injection enhanced the mRNA level of SOCS2 (*P* < 0.05, Figure 2(a)).  Figure 2(b) shows SOCS2 inhibited the release of FFA, but leptin reversed the effect (*P* < 0.05). There was no apparent change of TG level detected in only the Ad-SOCS2 and sh-SOCS2 treatment group (*P* > 0.05), while the TG level was elevated along with the incubation of leptin (*P* < 0.05, Figure 2(c)). The incorporation of [^14^C]-palmitate into adipose tissues was remarkably increased in the Ad-SOCS2 group but reduced after leptin injection, while sh-SOCS2 was opposite to Ad-SOCS2 (*P* < 0.05, Figure 2(d)).  Figure 2(e) shows that [^14^C]-palmitate oxidation to CO_2_ was lower in the Ad-SOCS2 treatment group compared with control group and was significantly elevated by sh-SOCS2, but the addition of leptin enhanced palmitate oxidation (*P* < 0.05). Forced expression of SOCS2 significantly downregulated the protein levels of CPT-1b and p-ACC (*P* < 0.05, Figures 2(g) and 2(h)), while increasing the FABP4 protein level (*P* < 0.05, Figure 2(f)). As shown in Figure 2(i), Ad-SOCS2 significantly decreased the leptin receptor level, the addition of leptin extenuated the reduction caused by Ad-SOCS2 and sh-SOCS2 has the opposite effect (*P* < 0.05).

### 3.3. SOCS2 Decreased Mitochondrial Fatty Acid Oxidation in Mouse Adipocytes

**Figure 3:**
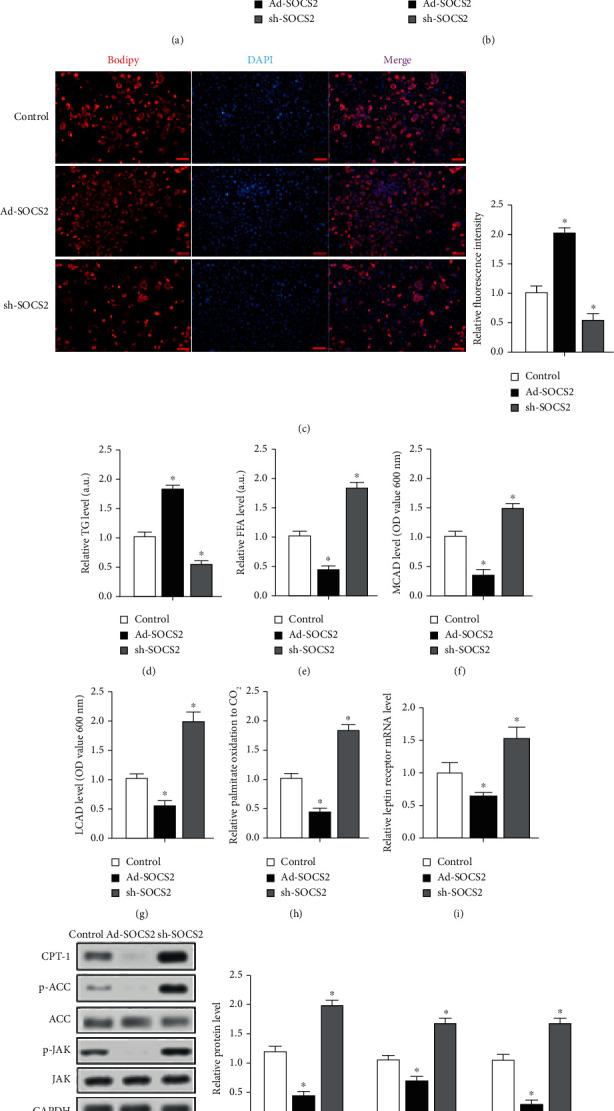
SOCS2 decreased mitochondrial fatty acid oxidation in mouse adipocytes. Four-week-old male mice were sacrificed, and stromal vascular cells were isolated from iWAT. (a) Protein expression level of SOCS2 after 48 h treatment with control vector, Ad-SOCS2, or sh-SOCS2 (*n* = 4); GAPDH was used as the loading control. (b) mRNA levels of SOCS1 and SOCS3 in iWAT after 48 h treatment with control vector, Ad-SOCS2, and sh-SOCS2 (*n* = 4). (c–j) Cells were transfected with different virus and induced to differentiation for 8 days and harvest for analysis (*n* = 4). (c) Bodipy staining; cells were carried out in mature adipocytes with respective treatment mentioned above. (d, e). Cellular TG content and FFA content quantification. (f, g) MCAD and LCAD protein levels. (h) Palmitate oxidation to CO_2_ was measured for 3 h (*n* = 4). (i) mRNA level of leptin receptor with qPCR. (j) Protein expression levels of CPT-1b, p-ACC, ACC1, p-JAK, and JAK; GAPDH was used as the loading control. Values are the means ± SD. ^∗^*P* < 0.05, ^∗∗^*P* < 0.01.

We then examined the influences of SOCS2 on fatty acid oxidation in mouse primary adipocytes. Four-week-old male mice were sacrificed, and stromal vascular cells were isolated from iWAT. Firstly, we evaluate the efficiency of Ad-SOCS2 or sh-SOCS2. After 48 h infection, the SOCS2 protein level increased in the Ad-SOCS2 group while decreased in the sh-SOCS2 group compared with the control group (*P* < 0.05, Figure 3(a)). Either Ad-SOCS2 or sh-SOCS2 had no effect on mRNA levels of SOCS1 and SOCS3 (*P* > 0.05, Figure 3(b)). After 8 days of adipogenic differentiation, overexpression of SOCS2 increased the accumulation of lipid drops and TG level (*P* < 0.05, Figures 3(c) and 3(d)), yet decreased the release of FFA (*P* < 0.05, Figure 3(e)). Moreover, the key enzymes of mitochondrial fatty acid oxidation medium-chain acyl-CoA dehydrogenase (MCAD) and long-chain acyl-CoA dehydrogenase (LCAD) levels were both significantly reduced in response to overexpression of SOCS2 and dramatically elevated by sh-SOCS2 (*P* < 0.05, Figures 3(f) and 3(g)). Ad-SOCS2 suppressed leptin receptor mRNA level and palmitate oxidation to CO_2_, and sh-SOCS2 had an opposite effect (*P* < 0.05, Figures 3(h) and 3(i)). Ad-SOCS2 significantly downregulated the protein levels of CPT-1b, p-ACC, and p-JAK, while sh-SOCS2 could promote phosphorylation of CPT1b, ACC, and JAK (*P* < 0.05, Figure 3(j)). These data implied that SOCS2 was a negative regulator of mitochondrial fatty acid oxidation.

### 3.4. SOCS2 Reduced Mitochondrial Respiratory Activity

**Figure 4:**
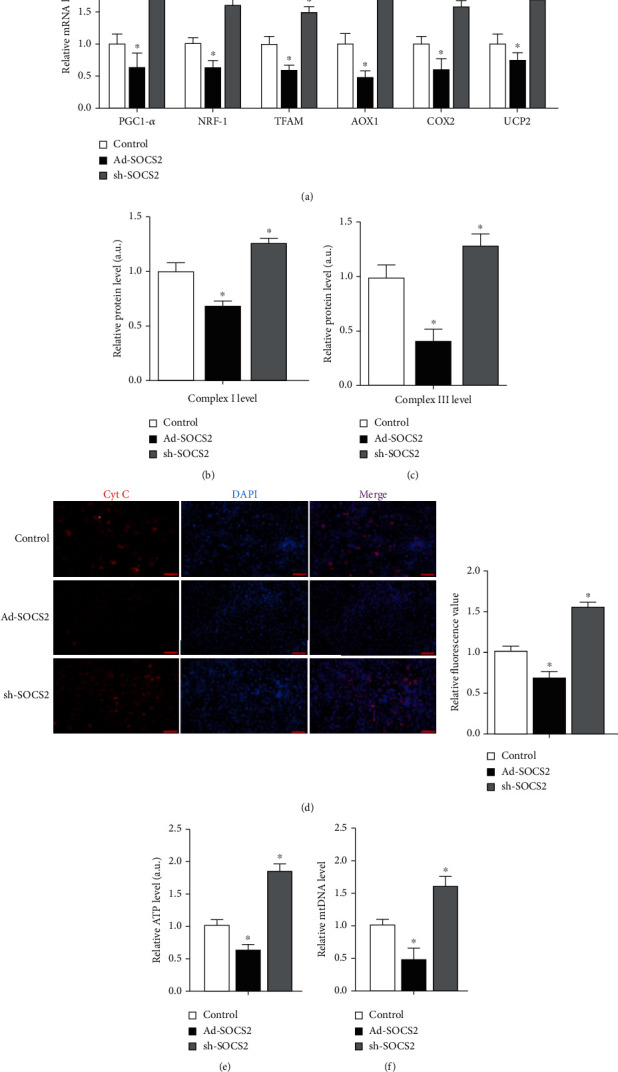
SOCS2 reduced mitochondrial respiratory activity. (a) mRNA expressions of the mitochondrial function genes after transfection with Ad-SOCS2 and sh-SOCS2 for 48 h (*n* = 4). (b, c) The activities of mitochondrial complexes I and III after vector delivery and differentiation for 8 days (*n* = 4). (d) Images of adipocytes Cyt C immunofluorescent staining after vector delivery and differentiation for 8 days (red). Scale bar: 100 *μ*m (*n* = 4). (e) ATP level after vector delivery and differentiation for 8 days (*n* = 4). (f) mtDNA copy number after vector delivery and differentiation for 8 days (*n* = 4). All the protein levels (b, c) were detected by the ELISA test. Values are the means ± SD. ^∗^*P* < 0.05, ^∗∗^*P* < 0.01.

Compared with the control group, mRNA levels of PGC-1*α*, nuclear respiratory factor 1 (NRF-1), mitochondrial transcription factor A (TFAM), aldehyde oxidase 1 (AOX1), cyclooxygenase-2 (COX2), and uncoupling protein 2 (UCP2) were all significantly downregulated in the Ad-SOCS2 group (*P* < 0.05, Figure 4(a)). Activities of mitochondrial complexes I and III were both reduced by SOCS2 overexpression (*P* < 0.05, Figures 4(b) and 4(c)). Cytochrome c (Cyt C) content was studied by immunofluorescent staining, and the fluorescence intensity showed Ad-SOCS2 also inhibited Cyt C expression, ATP level, and mitochondrial DNA (mtDNA) copy number, while they were increased by sh-SOCS2 (*P* < 0.05, Figures 4(d)–4(f)).

### 3.5. SOCS2 Inhibited Leptin-Induced Fatty Acid Oxidation in Mouse Adipocytes

**Figure 5:**
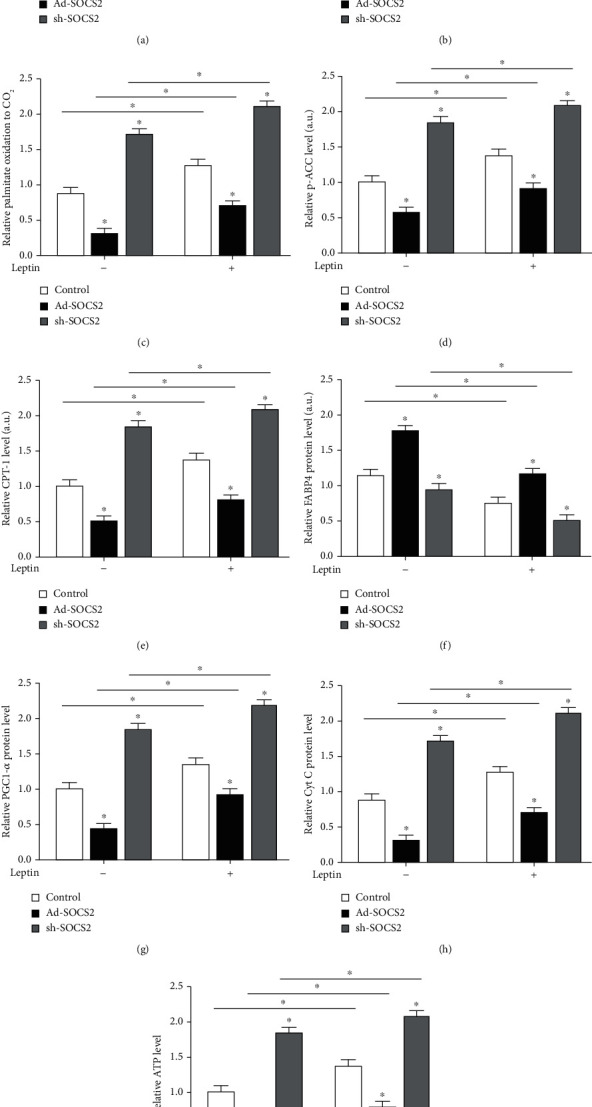
SOCS2 inhibited leptin-induced fatty acid oxidation in mouse adipocytes. Preadipocytes from mice iWAT were transfected with control vector, Ad-SOCS2, and sh-SOCS2 induced differentiation for 8 days, incubated with 100 nm leptin for 24 h on the last day before being harvested. (a) mRNA level of leptin receptor after transfection and leptin treatment (*n* = 4). (b) Cellular FFA content after transfection and leptin treatment (*n* = 4). (c) Fatty acid oxidation. Palmitate oxidation to CO_2_ was measured for 3 h after transfection and leptin treatment (*n* = 4). (d–h) p-ACC, CPT-1b, FABP4, PGC-1*α*, and Cyt C protein levels after transfection and leptin treatment (*n* = 4). All the protein levels were detected by the ELISA test. (i) ATP level after transfection and leptin treatment (*n* = 4). Values are the means ± SD. ^∗^*P* < 0.05, ^∗∗^*P* < 0.01.

As shown in Figure 3(i), Ad-SOCS2 suppressed leptin receptor at the mRNA level, but the addition of leptin had contrary function (*P* < 0.05, Figure 5(a)). Figures 5(b) and 5(c) show the addition of leptin elevated the release of FFA and [^14^C]-palmitate oxidation to CO_2_, but Ad-SOCS2 inhibited their levels and sh-SOCS2 had a promotion effect (*P* < 0.05). Increased p-ACC and CPT-1 levels by leptin were also reversed in the Ad-SOCS2 group (*P* < 0.05, Figures 5(d) and 5(e)). SOCS2 overexpression increased the protein level of FABP4, sh-SOCS2 could inhibit it, and cotreatment with leptin and SOCS2 attenuated this increased effect caused by Ad-SOCS2 (*P* < 0.05, Figure 5(f)). Leptin dramatically increased the levels of PGC-1*α*, Cyt C, and mitochondrial ATP, while Ad-SOCS2 had the reverse function; sh-SOCS2 could further increase their expression level on the basis of leptin treatment (*P* < 0.05, Figures 5(g)–5(i)). Consistent with the *in vivo* experiment, these data indicated SOCS2 inhibited leptin-induced fatty acid oxidation in mouse adipocytes.

### 3.6. SOCS2 Aggravated Oligomycin-Induced Mitochondrial Dysfunction

**Figure 6:**
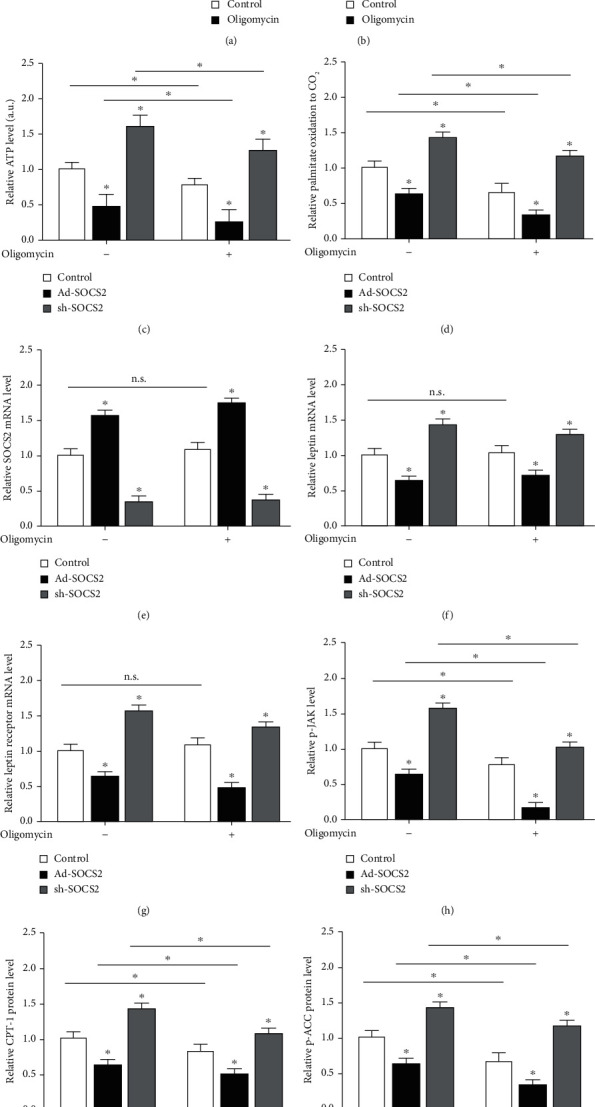
SOCS2 aggravated oligomycin-induced mitochondrial dysfunction. Preadipocytes from mice iWAT were transfected with control vector, Ad-SOCS2, and sh-SOCS2 induced differentiation for 8 days, incubated with 1 *μ*M oligomycin for 1 h before harvested. (a) Cell viability was detected by CCK-8 kit after 1 *μ*M oligomycin treatment 1 h (*n* = 4). (b) ATP level with 1 *μ*M oligomycin treatment for 1 h (*n* = 4). (c) ATP level after transfection and oligomycin incubation (*n* = 4). (d) Fatty acid oxidation. Palmitate oxidation to CO_2_ was measured for 3 h after transfection and oligomycin incubation (*n* = 4). (e–g) SCOS2 mRNA level, leptin, and its receptor mRNA level after transfection and treatment with oligomycin. (h) p-JAK level after transfection and treatment with oligomycin. (i, j) Protein expression levels of CPT-1b and p-ACC after transfection and treatment with 1 *μ*M oligomycin for 1 h (*n* = 4). All the protein levels (h–j) were detected by the ELISA test. Values are the means ± SD. ^∗^*P* < 0.05, ^∗∗^*P* < 0.01.

Oligomycin is an inhibitor of respiratory-chain phosphorylation which dramatically decreased the level of mitochondrial ATP, inhibiting the mitochondrial oxidation function. Oligomycin significantly reduced intracellular ATP levels (*P* < 0.05, Figure 6(a)) without affecting cell viability (*P* > 0.05, Figure 6(b)). SOCS2 overexpression exacerbated the impairment of mitochondrial respiration induced by oligomycin, whereas sh-SOCS2 elevated the ATP level and [^14^C]-palmitate oxidation to CO_2_, repairing impaired mitochondrial oxidation function (*P* < 0.05, Figures 6(c) and 6(d)). However, oligomycin did not have any effect on SOCS2, leptin, and its receptor mRNA level (*P* > 0.05, Figures 6(e)–6(g)). The suppressed p-JAK level after oligomycin treatment was further decreased by Ad-SOCS2 (*P* < 0.05, Figure 6(h)). Combining Figures 6(i) and 6(j), CPT-1b and p-ACC protein levels were both attenuated after oligomycin treatment and were accentuated in the Ad-SOCS2 group (*P* < 0.05). These results indicated SOCS2 had an aggravating effect on oligomycin-induced mitochondrial dysfunction.

### 3.7. JAK2 Signaling Pathway Was Involved in SOCS2 Regulation of Mitochondrial Fatty Acid Oxidation

**Figure 7:**
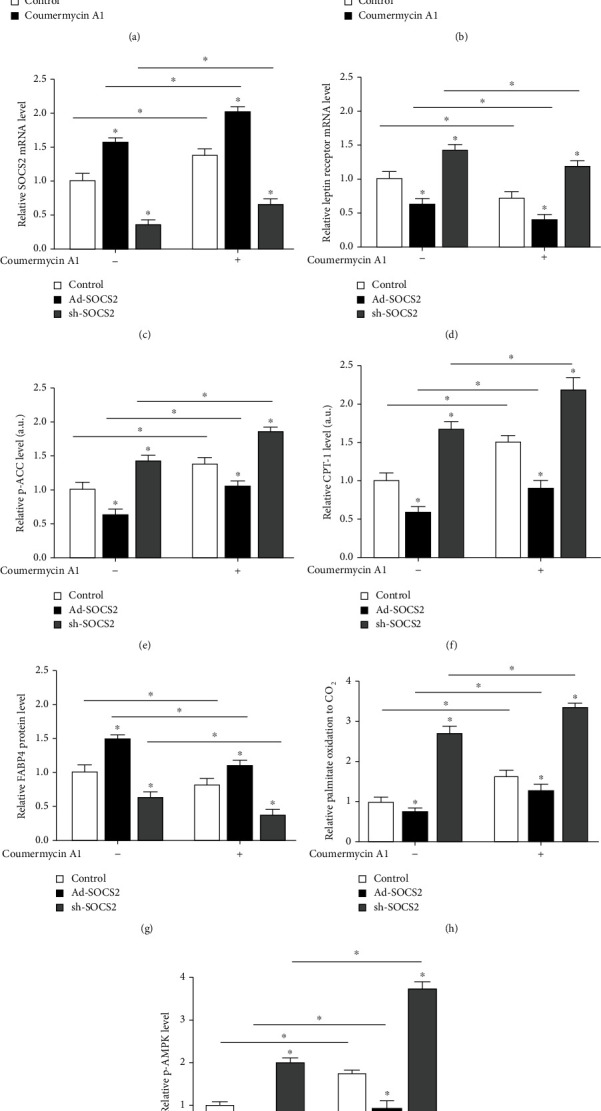
JAK2 signaling pathway was involved in SOCS2 regulation of mitochondrial fatty acid oxidation. The mice were treated with coumermycin A1, a JAK2 signal activator, and then were treated with saline, Ad-SOCS2, and sh-SOCS2. (a) p-JAK2 levels after 10 *μ*M coumermycin A1 treatment for 0 min, 10 min, 30 min, and 60 min (*n* = 4). (b) Fatty acid oxidation. Cells were treated with 10 *μ*M coumermycin A1 for 0 min, 10 min, 30 min, and 60 min, before palmitate oxidation to CO_2_ was measured (*n* = 4). (c) SOCS2 mRNA expression levels after transfection with Ad-SOCS2 and sh-SOCS2 and 10 *μ*M coumermycin A1 treatment for 30 min (*n* = 4). (d) Leptin receptor mRNA level after transfection with Ad-SOCS2 and sh-SOCS2 and 10 *μ*M coumermycin A1 treatment for 30 min (*n* = 4). (e–g) Protein levels of p-ACC, CPT-1b, and FABP4 after transfection with SOCS2 and 10 *μ*M coumermycin A1 treatment for 30 min (*n* = 4). (h) Fatty acid oxidation. Palmitate oxidation to CO_2_ was measured for 3 h after transfection with SOCS2 and 10 *μ*M coumermycin A1 treatment for 30 min (*n* = 4). (i) p-AMPK level after transfection with SOCS2 and 10 *μ*M coumermycin A1 treatment for 30 min (*n* = 4). All the protein levels (a, e–g, i) were detected by the ELISA test. Values are the means ± SD. ^∗^*P* < 0.05, ^∗∗^*P* < 0.01.

The level of p-JAK2 was increased especially after treated with coumermycin A1, which is a JAK2 signal activator (*P* < 0.05, Figure 7(a)). Coumermycin A1 increased [^14^C]-palmitate oxidation to CO_2_ (*P* < 0.05, Figure 7(b)).  Figure 7(c) indicates that coumermycin A1-induced-JAK2 signal activation increased the mRNA level of SCOS2 (*P* < 0.05), but reduced leptin receptor mRNA level (*P* < 0.05, Figure 7(d)). Then, we detected the expression of p-ACC and CPT-1b, as expected these two fatty acid oxidation marker genes expression increased significantly by coumermycin A1 treatment. But the increased p-ACC and CPT-1b levels induced by coumermycin A1 were reversed in Ad-SOCS2 group (*P* < 0.05, Figures 7(e) and 7(f)). The FABP4 protein level went down with the coumermycin A1 treatment but rose after overexpressing SOCS2 (*P* < 0.05, Figure 7(g)). Moreover, [^14^C]-palmitate oxidation to CO_2_ was upregulated by coumermycin A1 but reduced by Ad-SCOS2 and was elevated by sh-SOCS2 (*P* < 0.05, Figure 7(h)). These data proved that forced expression of SOCS2 abolished elevated mitochondrial function induced by the activation of JAK2.  Figure 7(i) further demonstrated that coumermycin A1 increased the p-AMPK level; however, Ad-SOCS2 downregulated it inversely. And cotreatment of sh-SOCS2 and coumermycin A1 could dramatically upregulate p-AMPK level (*P* < 0.05). Hence, we speculated SCOS2 inhibited mitochondrial function through the LepR/JAK2/AMPK signal pathway.

### 3.8. SOCS2 Reduced Mitochondrial Fatty Acid Oxidation through Inhibiting JAK2/AMPK Pathway

**Figure 8:**
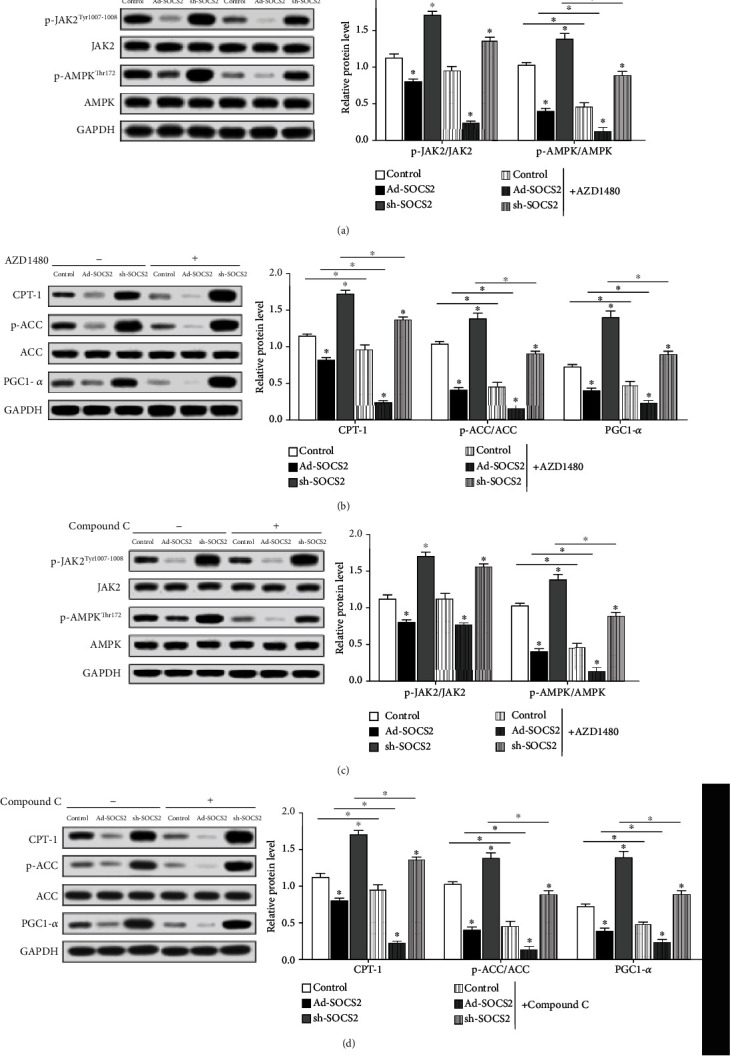
SOCS2 reduced mitochondrial fatty acid oxidation through inhibiting the JAK2/AMPK pathway. (a) Protein levels of p-JAK2^Tyr1007-1008^, JAK2, p-AMPK^Thr172^, and AMPK with Ad-SOCS2 or sh-SOCS2 transfection for 48 h and 1 nM AZD1480 (JAK2 inhibitor) incubation for 2 h (*n* = 4). (b) Protein expression levels of CPT-1b, p-ACC, ACC1, and PGC-1*α* with Ad-SOCS2 or sh-SOCS2 transfection for 48 h and 1 nM AZD1480 incubation for 2 h (*n* = 4). (c) Protein expression levels of p-JAK2^Tyr1007-1008^, JAK2, p-AMPK^Thr172^, and AMPK with Ad-SOCS2 or sh-SOCS2 transfection for 48 h and 1 nM Compound C (AMPK inhibitor) incubation for 1 h (*n* = 4). (d) Protein expression levels of CPT-1b, p-ACC, ACC1, and PGC-1*α* with Ad-SOCS2 or sh-SOCS2 transfection for 48 h and 1 nM Compound C incubation for 1 h (*n* = 4). GAPDH was used as the loading control in western blot. Values are the means ± SD. ^∗^*P* < 0.05, ^∗∗^*P* < 0.01.

To further confirm the molecular mechanism of SOCS2 on mitochondrial fatty acid oxidation, we measured the phosphorylation levels of JAK2 and AMPK.  Figure 8(a) shows overexpression of SOCS2 reduced p-JAK2 and p-AMPK levels; sh-SOCS2 significantly increases their levels (*P* < 0.05). Additionally, protein levels of CPT-1b, p-ACC/ACC1, and PGC-1*α* were also elevated by Ad-SOCS2 (*P* < 0.05, Figure 8(b)). Treatment with JAK2-specific inhibitor AZD1480 decreased JAK2 phosphorylation and also reduced AMPK phosphorylation (*P* < 0.05, Figure 8(a)). Conversely, overexpression of SOCS2 inhibited mitochondrial fatty acid oxidation (*P* < 0.05, Figure 8(b)) along with the reduced p-JAK2 level. Similarly, the AMPK-specific inhibitor Compound C decreased AMPK phosphorylation but could not reduce JAK2 phosphorylation (*P* < 0.05, Figure 8(c)). Still, the levels of CPT-1b, p-ACC/ACC1, and PGC-1*α* were decreased by Ad-SOCS2 and elevated by sh-SOCS2 as shown in Figure 8(d) (*P* < 0.05).

## 4. Discussion

Our research proved that SOCS2 is the downregulator of leptin signaling with the biochemical consequence of fatty acid oxidation inhibition. SOCS proteins are negative regulators of cytokine signaling that function primarily at the receptor level. SOCS2, as an intracellular protein induced by cytokines and hormones, could modulate the immune response, neural development, neurogenesis, and neurotrophic pathways [11, 35]. Many studies have proved that SOCS2 has complex biological functions [19, 36, 37], such as SOCS2 which has an inhibitory effect on GH, interferon, and leptin signaling. It has been reported that SOCS3 can be induced by high-dose leptin [38]. The complicated interaction between SOCS2 with LepR implied a role of SOCS2 in inhibiting recruitment of downstream signaling moieties [19]. Multiple studies have shown that leptin can promote the expression of SOCS2 [36, 39]. Our research also proves that SOCS2 is a key regulatory molecule downstream of leptin and could be elevated by leptin. However, Lavens et al. showed that the conservative Y985 and Y1077 patterns in the cytoplasmic domain of leptin receptor have specific binding to CIS [19]. SOCS2 only interacts with the Y1077 motif, but has a higher binding affinity, and can interfere with the recruitment of CIS and STAT5a at this site. In addition, although SOCS2 does not associate with Y985 of the leptin receptor, SOCS2 can prevent the interaction of CIS with this position and inhibit the binding of leptin receptor to the target gene. And in Figures 2(i) and 3(i) of our research, data also indicated that overexpression of SOCS2 can significantly inhibit the expression of Leptin receptor in adipocytes; this is consistent with those previous studies. We demonstrate this negative feedback mechanism causes the expression of SOCS2 to be increased by leptin, and instead, SOCS2 inhibits the activity of leptin receptor, which weakens the promotion of leptin on fatty acid oxidation in adipocytes.

The data of our research indicated that leptin could promote lipolysis. Leptin directly inhibits intracellular lipid concentrations by reducing fatty acid, triglyceride synthesis, and concomitantly increasing lipid oxidation [39, 40]. Studies have shown that exogenous addition of leptin can lead to inhibition of lipogenesis, increased hydrolysis of triglycerides, and increased oxidation of fatty acids and glucose, and activation of central leptin receptors also contributes to the development of catabolic states in adipocytes [41, 42]. Our data also show that FFA in plasma are elevated by leptin, and lipolysis-related genes PGC1*α*, CPT-1, p-ACC, FAT can be upregulated by leptin. This implies that leptin could promote lipolysis. Leptin increased FAO by stimulating the activity of CPT-1 and inhibiting activity of ACC, respectively [43, 44]. Inhibition of ACC will thus block fatty acid synthesis and favor mitochondrial fatty acid uptake and oxidation, resulting in lower intracellular fatty acid and triglyceride concentrations [45]. Recent research shows a novel mechanism of leptin-induced FAO in the muscle tissue, in which leptin stimulated fatty acid uptake to enhance fatty acid oxidation via AMPK activation in both mouse muscle and cardiac myocytes [46]. In our results, we also confirmed that leptin suppressed fatty acid synthesis and increased SOCS2 expression in the adipose tissue.

Fatty acids are the main components of various types of metabolic activities, and they are essential for the various cellular functions of mammals. The body can maintain the supply of cellular fatty acids through various mechanisms. Cellular fatty acid levels are highly dynamic and depend on the metabolic needs of cells and organisms, which may be affected by disease, fasting and feeding, exercise, and temperature [47]. These factors determine the pathway of fatty acid utilization, including mitochondrial FAO (*β*-oxidation) as an energy source or TG storage in LD. Studies have shown that FAO is required for cold-induced heat generation in BAT and oxidative stress and inflammation induced by a high-fat diet in WAT [48, 49]. In addition, FAO is necessary to maintain active and resting BAT and thermogenesis program [50]. Defects in FAO are thought to be related to obesity-related metabolic disorders. There is evidence that during obesity, the decreased ability of FAO in humans and rodents leads to lipid accumulation and lipid toxicity [51, 52]. Some studies have shown that increasing FAO can effectively combat obesity and insulin resistance [53, 54]. In our study, we demonstrated that silencing SOCS2 can promote FAO by promoting the LepR/JAK2/AMPK signaling pathway in mouse adipocytes and maintain the lipid metabolism activity of adipocytes.

**Figure 9:**
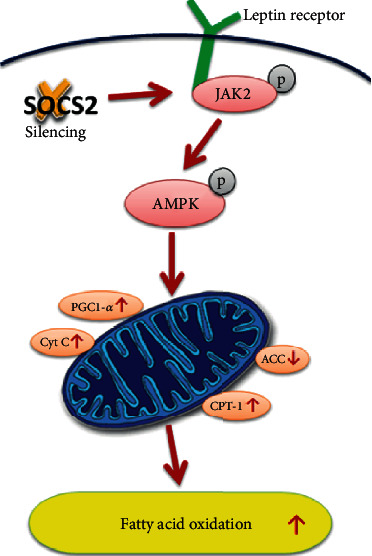
Summary of SOCS2 in the regulation of mitochondrial fatty acid oxidation via LepR/JAK2/AMPK signaling pathway. SOCS2 and leptin play opposite roles in mitochondrial fatty acid oxidation.

Taken together, our data indicated that silencing of SOCS2 promoted mitochondrial fatty acid oxidation through elevating phosphorylation levels of JAK2 and AMPK, accompanied with decreased expression of leptin receptor (Figure 9). These results may provide a new insight into the molecular mechanism by which fatty acid oxidation is regulated by SOCS2 and implicate a new therapy against obesity and related metabolic syndrome.

## Data Availability

The data used to support the findings of this study are available from the corresponding authors upon request.

## Abbreviations

SOCS2: Suppressor of cytokine signaling 2
LepR: Leptin receptor
GH: Growth hormone
PGC-1*α*: Peroxisome proliferator-activated receptor gamma coactivator 1-alpha
CPT-1b: Carnitine palmitoyl transferase I-b
FAT: Fatty acid translocase
ACC1: Acetyl-CoA carboxylase 1
FABP4: Fatty acid-binding protein 4
FATP1: Fatty acid transport protein 1
FAO: Fatty acid oxidation
NRF-1: Nuclear respiratory factor 1
TFAM: Mitochondrial transcription factor A
AOX1: Aldehyde oxidase 1
COX2: Cyclooxygenase 2
UCP2: Uncoupling protein 2
MCAD: Medium-chain acyl-CoA dehydrogenase
LCAD: Long-chain acyl-CoA dehydrogenase
Cyt C: Cytochrome c
JAK/STAT: Janus kinase/signal transducers and activators of transcription
AMPK: AMP-activated protein kinase
iWAT: Inguinal white adipose tissue
TG: Triglycerides.

## References

[1] Yoshimura A., Naka T., Kubo M. (2007). SOCS proteins, cytokine signalling and immune regulation. *Nature Reviews Immunology*.

[2] Liu Z., Gan L., Yang X., Zhang Z., Sun C. (2014). Hydrodynamic tail vein injection of SOCS3 eukaryotic expression vector in vivo promoted liver lipid metabolism and hepatocyte apoptosis in mouse. *Biochemistry and Cell Biology*.

[3] Liu Z., Gan L., Zhou Z., Jin W., Sun C. (2015). SOCS3 promotes inflammation and apoptosis via inhibiting JAK2/STAT3 signaling pathway in 3T3-L1 adipocyte. *Immunobiology*.

[4] Siegrist-Kaiser C. A., Pauli V., Juge-Aubry C. E. (1997). Direct effects of leptin on brown and white adipose tissue. *Journal of Clinical Investigation*.

[5] Krebs D. L., Hilton D. J. (2001). SOCS Proteins: Negative Regulators of Cytokine Signaling. *Stem Cells*.

[6] Yang H. L., Sun C., Sun C., Qi R. L. (2012). Effect of suppressor of cytokine signaling 2 (SOCS2) on fat metabolism induced by growth hormone (GH) in porcine primary adipocyte. *Molecular Biology Reports*.

[7] Miller M. E., Michaylira C. Z., Simmons J. G. (2004). Suppressor of cytokine signaling-2: a growth hormone-inducible inhibitor of intestinal epithelial cell proliferation. *Gastroenterology*.

[8] Ricobautista E., Floresmorales A., Fernandezperez L. (2006). Suppressor of cytokine signaling (SOCS) 2, a protein with multiple functions. *Cytokine & Growth Factor Reviews*.

[9] Haan S. (2016). SOCS2 physiological and pathological functions. *Frontiers in Bioscience*.

[10] Das R., Gregory P. A., Fernandes R. C. (2017). MicroRNA-194 Promotes Prostate Cancer Metastasis by Inhibiting SOCS2. *Cancer Research*.

[11] Nirschl C. J., Suárez-Fariñas M., Izar B. (2017). IFN*γ*-Dependent Tissue-Immune Homeostasis Is Co-opted in the Tumor Microenvironment. *Cell*.

[12] Leshan R. L., Greenwald-Yarnell M., Patterson C. M., Gonzalez I. E., Myers M. G. (2012). Leptin action through hypothalamic nitric oxide synthase-1-expressing neurons controls energy balance. *Nature Medicine*.

[13] Cui H., López M., Rahmouni K. (2017). The cellular and molecular bases of leptin and ghrelin resistance in obesity. *Nature Reviews Endocrinology*.

[14] Dodington D. W., Desai H. R., Woo M. (2018). JAK/STAT - Emerging Players in Metabolism. *Trends in Endocrinology and Metabolism*.

[15] Howard J. K., Flier J. S. (2006). Attenuation of leptin and insulin signaling by SOCS proteins. *Trends in Endocrinology & Metabolism*.

[16] Jorgensen S. B., O’Neill H. M., Sylow L. (2012). Deletion of Skeletal Muscle SOCS3 Prevents Insulin Resistance in Obesity. *Diabetes*.

[17] Steinberg G. R., McAinch A. J., Chen M. B. (2006). The Suppressor of Cytokine Signaling 3 Inhibits Leptin Activation of AMP-Kinase in Cultured Skeletal Muscle of Obese Humans. *The Journal of Clinical Endocrinology & Metabolism*.

[18] Tom R. Z., Garcia-Roves P. M., Sjögren R. J. O. (2014). Effects of AMPK Activation on Insulin Sensitivity and Metabolism in Leptin-Deficientob/obMice. *Diabetes*.

[19] Lavens D. (2006). A complex interaction pattern of CIS and SOCS2 with the leptin receptor. *Journal of Cell Science*.

[20] Yu X., McCorkle S., Wang M. (2004). Leptinomimetic effects of the AMP kinase activator AICAR in leptin-resistant rats: prevention of diabetes and ectopic lipid deposition. *Diabetologia*.

[21] Tang X., Li J., Xiang W. (2016). Metformin increases hepatic leptin receptor and decreases steatosis in mice. *Journal of Endocrinology*.

[22] Wajner M., Amaral A. U. (2016). Mitochondrial dysfunction in fatty acid oxidation disorders: insights from human and animal studies. *Bioscience Reports*.

[23] Wanders R. J. A., Ruiter J. P. N., IJlst L., Waterham H. R., Houten S. M. (2010). The enzymology of mitochondrial fatty acid beta-oxidation and its application to follow-up analysis of positive neonatal screening results. *Journal of Inherited Metabolic Disease*.

[24] Bastin J. (2014). Regulation of mitochondrial fatty acid *β*-oxidation in human: what can we learn from inborn fatty acid *β*-oxidation deficiencies?. *Biochimie*.

[25] Gan L., Liu Z., Chen Y. (2016). *α*-MSH and Foxc2 promote fatty acid oxidation through C/EBP*β* negative transcription in mice adipose tissue. *Scientific Reports*.

[26] Houten S. M., Violante S., Ventura F. V., Wanders R. J. A. (2016). The Biochemistry and Physiology of Mitochondrial Fatty Acid *β*-Oxidation and Its Genetic Disorders. *Annual Review of Physiology*.

[27] Gan L., Liu Z., Zhang Z., Yang X., Liu J., Sun C. (2014). SOCS2 inhibited mitochondria biogenesis via inhibiting p38 MAPK/ATF2 pathway in C2C12 cells. *Molecular Biology Reports*.

[28] Liu Z., Gan L., Luo D., Sun C. (2017). Melatonin promotes circadian rhythm-induced proliferation through Clock/histone deacetylase 3/c-Myc interaction in mouse adipose tissue. *Journal of Pineal Research*.

[29] Liu Z., Gan L., Zhang T., Ren Q., Sun C. (2018). Melatonin alleviates adipose inflammation through elevating *α*-ketoglutarate and diverting adipose-derived exosomes to macrophages in mice. *Journal of Pineal Research*.

[30] Sebastián D., Guitart M., García-Martínez C. (2009). Novel role of FATP1 in mitochondrial fatty acid oxidation in skeletal muscle cells. *Journal of Lipid Research*.

[31] Gan L., Liu Z., Cao W., Zhang Z., Sun C. (2015). FABP4 reversed the regulation of leptin on mitochondrial fatty acid oxidation in mice adipocytes. *Scientific Reports*.

[32] Buettner C., Muse E. D., Cheng A. (2008). Leptin controls adipose tissue lipogenesis via central, STAT3-independent mechanisms. *Nature Medicine.*.

[33] Liu Z., Gan L., Wu T. (2016). Adiponectin reduces ER stress-induced apoptosis through PPAR _*α*_ transcriptional regulation of ATF2 in mouse adipose. *Cell Death & Disease*.

[34] Liu Z., Gan L., Xu Y. (2017). Melatonin alleviates inflammasome-induced pyroptosis through inhibiting NF-*κ*B/GSDMD signal in mice adipose tissue. *Journal of Pineal Research*.

[35] Brant F., Miranda A. S., Esper L. (2016). Suppressor of cytokine signaling 2 modulates the immune response profile and development of experimental cerebral malaria. *Brain, Behavior, and Immunity*.

[36] Bolamperti S., Mrak E., Moro G. L. (2013). 17*β*-Estradiol positively modulates growth hormone signaling through the reduction of SOCS2 negative feedback in human osteoblasts. *Bone*.

[37] Terán-Cabanillas E., Hernández J. (2017). Role of Leptin and SOCS3 in Inhibiting the Type I Interferon Response During Obesity. *Inflammation*.

[38] Higuchi H., Hasegawa A., Yamaguchi T. (2005). Transcriptional Regulation of Neuronal Genes and Its Effect on Neural Functions: Transcriptional Regulation of Neuropeptide Y Gene by Leptin and Its Effect on Feeding. *Journal of Pharmacological Sciences*.

[39] Simonds S. E., Pryor J. T., Ravussin E. (2014). Leptin Mediates the Increase in Blood Pressure Associated with Obesity. *Cell*.

[40] Ruud J., Brüning J. C. (2015). Light on leptin link to lipolysis. *Nature*.

[41] Harris R. B. S. (2014). Direct and indirect effects of leptin on adipocyte metabolism. *Biochimica et Biophysica Acta (BBA) - Molecular Basis of Disease*.

[42] Zeng W., Pirzgalska R. M., Pereira M. M. A. (2015). Sympathetic Neuro-adipose Connections Mediate Leptin-Driven Lipolysis. *Cell*.

[43] Sloan C., Tuinei J., Nemetz K. (2011). Central leptin signaling is required to normalize myocardial fatty acid oxidation rates in caloric-restricted ob/ob mice. *Diabetes*.

[44] Wei J., Tong L. (2015). Crystal structure of the 500-kDa yeast acetyl-CoA carboxylase holoenzyme dimer. *Nature*.

[45] Jeon S.-M., Chandel N. S., Hay N. (2012). AMPK regulates NADPH homeostasis to promote tumour cell survival during energy stress. *Nature*.

[46] Momken I., Chabowski A., Dirkx E. (2017). A new leptin-mediated mechanism for stimulating fatty acid oxidation: a pivotal role for sarcolemmal FAT/CD36. *Biochemical Journal*.

[47] Schrauwen P., van Marken Lichtenbelt W. D. (2016). Combatting type 2 diabetes by turning up the heat. *Diabetologia*.

[48] Lee J., Ellis J. M., Wolfgang M. J. (2015). Adipose Fatty Acid Oxidation Is Required for Thermogenesis and Potentiates Oxidative Stress-Induced Inflammation. *Cell Reports*.

[49] Steensels S., Ersoy B. A. (2019). Fatty acid activation in thermogenic adipose tissue. *Biochimica et Biophysica Acta (BBA) - Molecular and Cell Biology of Lipids*.

[50] Gonzalez-Hurtado E., Lee J., Choi J., Wolfgang M. J. (2018). Fatty acid oxidation is required for active and quiescent brown adipose tissue maintenance and thermogenic programing. *Molecular Metabolism.*.

[51] Boyle K. E., Zheng D., Anderson E. J., Neufer P. D., Houmard J. A. (2012). Mitochondrial lipid oxidation is impaired in cultured myotubes from obese humans. *International Journal of Obesity.*.

[52] Ritov V. B., Menshikova E. V., He J., Ferrell R. E., Goodpaster B. H., Kelley D. E. (2004). Deficiency of Subsarcolemmal Mitochondria in Obesity and Type 2 Diabetes. *Diabetes*.

[53] Herrero L., Rubí B., Sebastián D. (2005). Alteration of the malonyl-CoA/Carnitine palmitoyltransferase I interaction in the-cell impairs glucose-Induced Insulin Secretion. *Diabetes*.

[54] Sebastián D., Herrero L., Serra D., Asins G., Hegardt F. G. (2007). CPT I overexpression protects L6E9 muscle cells from fatty acid-induced insulin resistance. *American Journal of Physiology-Endocrinology and Metabolism.*.

